# Strengthening Water Quality Monitoring Following Cyclones Idai and Kenneth in Mozambique

**DOI:** 10.4236/jwarp.2025.172006

**Published:** 2025-02

**Authors:** Anu Rajasingham, Travis Brown, Arminda Macuamule, Felisberto Lúcio, Garbaldino Zeca, Jorge Matola, Didier Monteiro, Tomohiko Morita, Alexia Couture, Albert Reichert, Pierre-Yves Oger, Chris Cormency, Thomas Handzel

**Affiliations:** 1 Global Public Health Emergencies Branch, US Centers for Disease Control and Prevention, Atlanta, USA; 2 Waterborne Disease Prevention Branch, US Centers for Disease Control and Prevention, Atlanta, USA; 3 Sofala Direcção Provincial de Saúde, Beira, Mozambique; 4 Autoridade Reguladora de Água, Beira, Mozambique; 5 Fundo de Investimento e Património do Abastecimento de Água 5, Beira, Mozambique; 6 United Nations Children’s Fund, Mozambique 6, Maputo, Mozambique; 7 Bureau for Humanitarian Assistance, US Agency for International Development, Washington DC, USA; 8 United Nations Children’s Fund, New York City, USA

**Keywords:** Water Quality Monitoring, Emergency Response, Cholera Outbreak Response, Chlorination

## Abstract

In early 2019, Mozambique was struck by two cyclones, Cyclone Idai in Sofala Province and Cyclone Kenneth in Cabo Delgado Province. Outbreaks of cholera were declared soon after both cyclones in Beira and Pemba cities. In response to the emergencies and outbreaks, government and humanitarian partners collaborated to create a mobile phone based water quality monitoring program to monitor daily free residual chlorine (FRC) levels in the piped network in both locations and at accommodation centers created for internally displaced persons in Beira. Overall, 87% of the 1080 samples from the piped network in Beira had detectable FRC and at accommodation centers, 73% of the 179 samples collected had detectable FRC. In Pemba, 64% of the 114 total samples collected had detectable FRC. Data from the water quality monitoring programs allowed for the identification of trends that helped increase the effectiveness of the response, including identifying areas where chlorination could be strengthened with the installation of booster chlorinators, issues with the consistency of daily chlorine treatment, and sites where water availability was limited. The water quality monitoring activities were a result of productive collaboration and could be replicated after similar emergencies in cholera endemic areas to prevent and control outbreaks.

## 1. Introduction

Providing access to safe drinking water is critical during humanitarian emergencies and outbreaks of waterborne diseases such as cholera [1] [2]. Chlorination of drinking water supplies is a commonly utilized method to provide safe water due to the cost-effectiveness, efficacy in inactivating bacterial and viral pathogens, ability to provide residual protection, and ease of monitoring by testing for free residual chlorine (FRC) [3]. During outbreaks and emergencies, the recommended FRC level for drinking water is 0.5 mg/L at the point-of-use, 1.0 mg/L at tapstands, and 2.0 mg/L at water trucks during filling [4]. Routine monitoring of FRC levels is essential to ensure the continued provision of safe water [5].

Mozambique was struck by two destructive cyclones within six weeks of each other, Cyclone Idai and Cyclone Kenneth. Cyclone Idai made landfall over Mozambique’s city of Beira, Sofala Province, on March 15^th^, 2019. It is estimated that in Sofala province, 387,165 persons were affected and that 78,432 internally displaced persons were forced to seek shelter in accommodation centers that were created by government and humanitarian partners as temporary housing for displaced persons [6]. The piped water network servicing Beira and neighboring Dondo (hereafter referred to as the Beira system) was severely impacted by the cyclone; and water provision was halted for approximately one week. Humanitarian partners supported the water utility, by restoring the power supply to the water treatment plant, fixing network infrastructure, installing emergency water treatment units, trucking water to affected areas, and providing water treatment chemicals. Cholera is endemic to Sofala province, and an outbreak was declared soon after the cyclone on March 27^th^. The Ministry of Health reported the total number of cholera cases in the province from the outbreak to be 6739 [7].

On April 25^th^, Cyclone Kenneth made landfall on the northernmost coast of Cabo Delgado province in Mozambique. Estimates suggest that a total of 254,750 people were affected by the cyclone and 3180 people were displaced and in accommodation centers. An outbreak of cholera was declared on May 2^nd^ in Pemba city and a total of 284 cases of cholera were reported in Cabo Delgado Province [8].

The water utility in Mozambique, Fundo de Investimento e Património do Abastecimento de Água (FIPAG), estimated that the Beira piped network system provides water for 60% of the population with over 60,000 connections and 240 community tapstands. Routine water quality monitoring of the piped networks in Beira was affected and limited immediately following Cyclone Idai. Thus, it was paramount to restart water quality monitoring activities following the cyclone and the cholera outbreak. In response to the emergency and outbreak, the Autoridade Reguladora de Água (AURA), the government sanctioned water quality regulatory authority, proposed to scale-up their water quality monitoring to conduct daily monitoring with support from the Sofala Provincial Health Department, Direcção Provincial de Saúde (DPS). The objective of the emergency water quality monitoring program was to ensure that drinking water supplies serving Beira met the minimum water quality standards during a cholera outbreak. AURA and DPS proposed to monitor both piped network water as well as water trucked and piped into the accommodation centers created after the cyclone. The US Centers for Disease Control and Prevention (CDC) and the United Nations Children’s Fund (UNICEF) supported this effort with the development of a mobile phone-based water quality monitoring and reporting program.

Based on the success of the program in Beira, soon after Cyclone Kenneth landed in Pemba, a similar water quality monitoring program was implemented in Pemba where FIPAG estimated the piped network provided 30% of the population its drinking water. Our report details the water quality monitoring programs implemented in Beira and Pemba and highlights the programs’ ability to collect timely water quality data to improve chlorination through the piped network and at accommodation centers during both humanitarian and cholera outbreak responses.

## 2. Methods

To ensure that drinking water provided through the piped network and at the accommodation centers met the minimum standard recommended during a cholera outbreak, FRC, was the priority water quality parameter included in the program. In Beira, FRC was tested using the N,N-diethyl-phenylenediamine (DPD) colorimetric method with Water-I.D. comparators (Eggenstein, Germany). In Pemba, FRC was tested using DPD colorimetric method with a Hach chlorine color disc kit (Colorado, United States). One type of kit was used in each location to ensure comparability of results over time for Beira and Pemba respectively. Trainings were conducted at the start of the monitoring programs to ensure technicians were comfortable with the water testing kits. In Beira and Dondo, at the start of the program, 41 sentinel sampling points in the piped water network were included. These points were in strategic locations, including near the water treatment plant and before and after piped network distribution centers where chlorine boosting occurred. CRA selected these sentinel points with FIPAG the water utility to ensure their suitability for monitoring the piped network. In addition, sampling points were established in all open accommodation centers in Beira and Dondo. In Pemba, 30 sentinel points in the piped network were included at similar locations, such as near the water treatment plant and before and after chlorine boosting sites. However, in Pemba, the piped network only supplied water to specific areas of the city each day, on a rotational basis, meaning that water was available in different areas of the city depending on the day of the week. Thus points in Pemba were monitored only when water was reported to be available by FIPAG.

AURA and DPS hired enumerators for this activity. CDC created a mobile data collection survey form for this activity using the data collection platform, ONA (Nairobi, Kenya), and the form was housed on UNICEF’s ONA server. CDC trained all technician/enumerators and supervisors on mobile data collection using ONA on Android phones. Following the training, data was collected and shared daily with all partners involved to inform the utility’s water treatment processes. In addition, the data was analyzed and mapped three times per week and shared with all national cholera taskforce members in addition to the partners involved with this program. Spatial results were produced using Quantum GIS (QGIS) software version 2.18. After three weeks of initial data analysis and mapping in Beira, AURA and DPS were trained on reporting, mapping, and sharing data. In Pemba, AURA was trained after one week of data collection. Additional data analysis and visualization was conducted using R Studio version 1.1.423 [9].

## 3. Results

Water quality monitoring data was collected and analyzed daily and summarized three times per week both quantitatively and spatially. This information was sent to all emergency collaborators on this project; and AURA, DPS, and FIPAG met regularly to discuss results and plan remediation strategies based on results. Water quality monitoring data from Beira will be described separately from data collected from Pemba.

### 3.1. Beira

**Figure 1.**
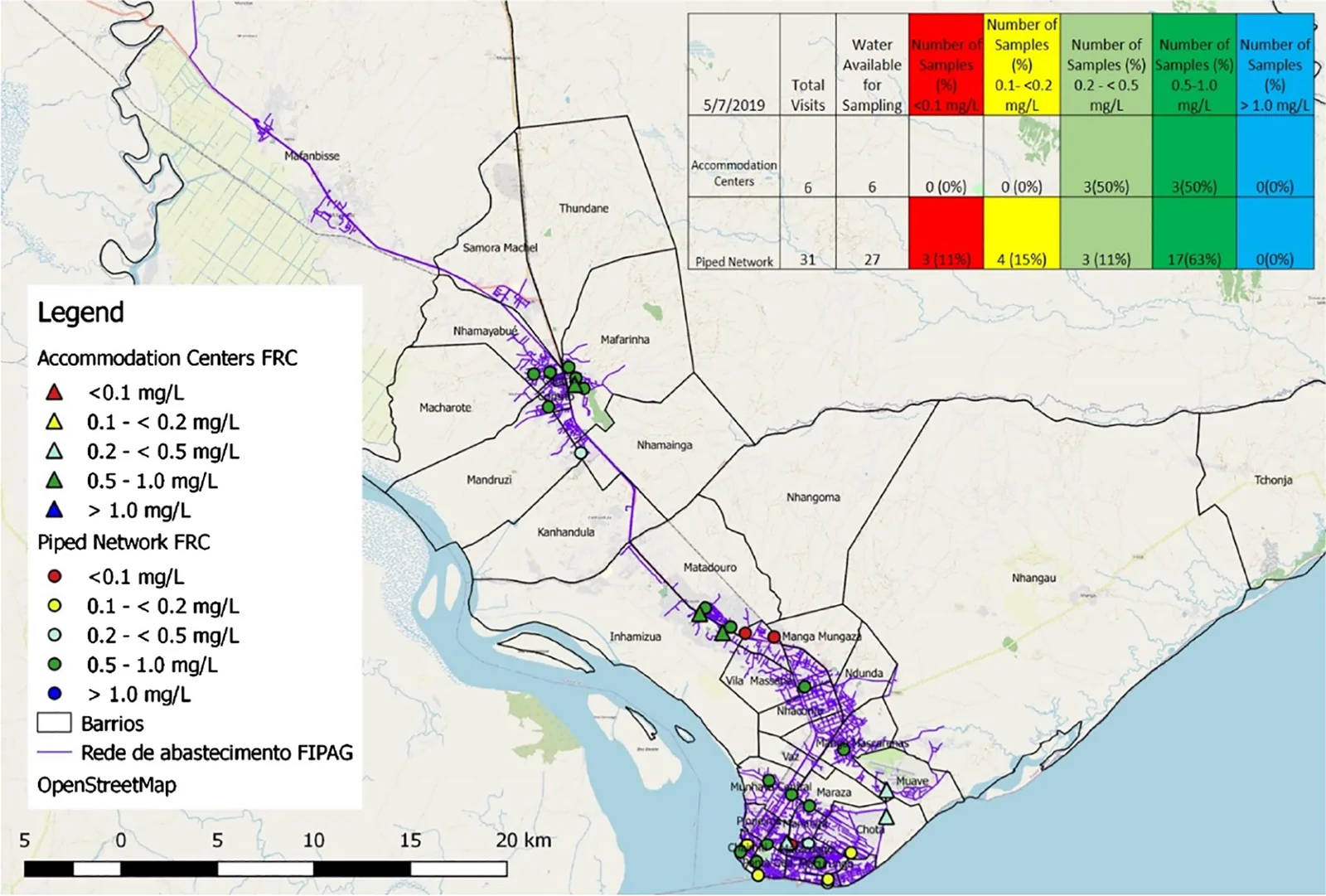
Example daily water quality monitoring report by AURA, DPS, UNICEF, and CDC shows summary and spatial distribution of FRC in Beira on May 7^th^, 2019. *Rede de Abastecimento means piped network in English.

In Beira, water quality monitoring data collection began on May 4^th^, 2019. An example figure presented to partners is shown in Figure 1. Overall, between May 4^th^ and June 14^th^, 87% of the 1080 samples collected from the 41 sampling points on the piped network, had FRC ≥ 0.1 mg/L and 46% of the samples were in the recommended FRC range: 0.5 – 1.0 mg/L. From the accommodation centers, 73% of the 177 samples collected had detectable FRC and 39% of the samples were in the recommended range. The median FRC seen in the piped network was 0.4 mg/L, and the range was from 0.0 mg/L - 1.9 mg/L. The median FRC observed in the accommodation centers was 0.3 mg/L and the range was 0.0 mg/L - 1.0 mg/L. Table 1 includes a weekly summary of all data collected between May 4^th^ to June 14^th^ from the piped network and accommodation centers in Beira.

**Figure 2.**
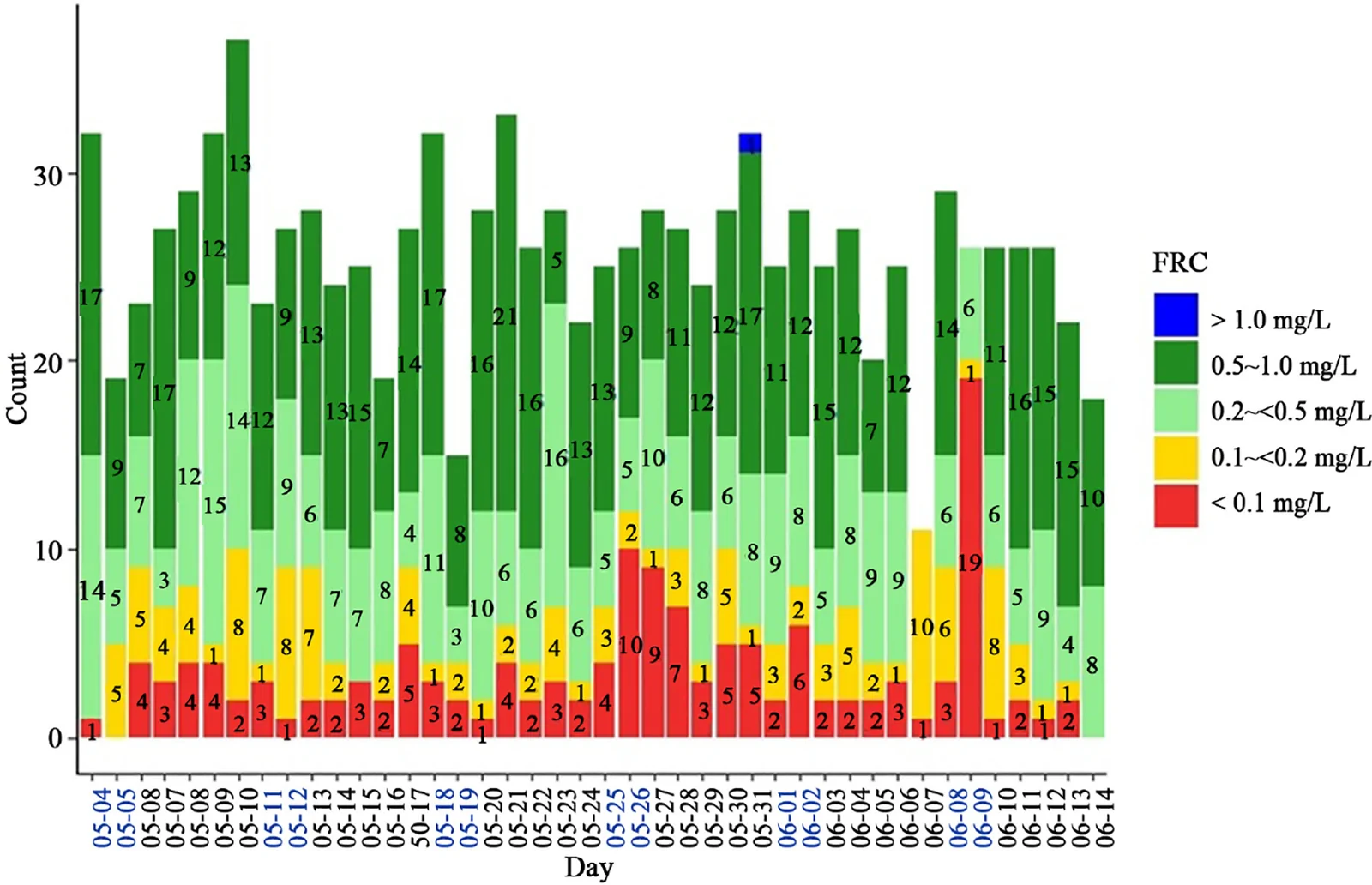
Free residual chlorine levels detected in the piped network by day and week in Beira, Mozambique. *dates in blue are weekend dates.

**Figure 3.**
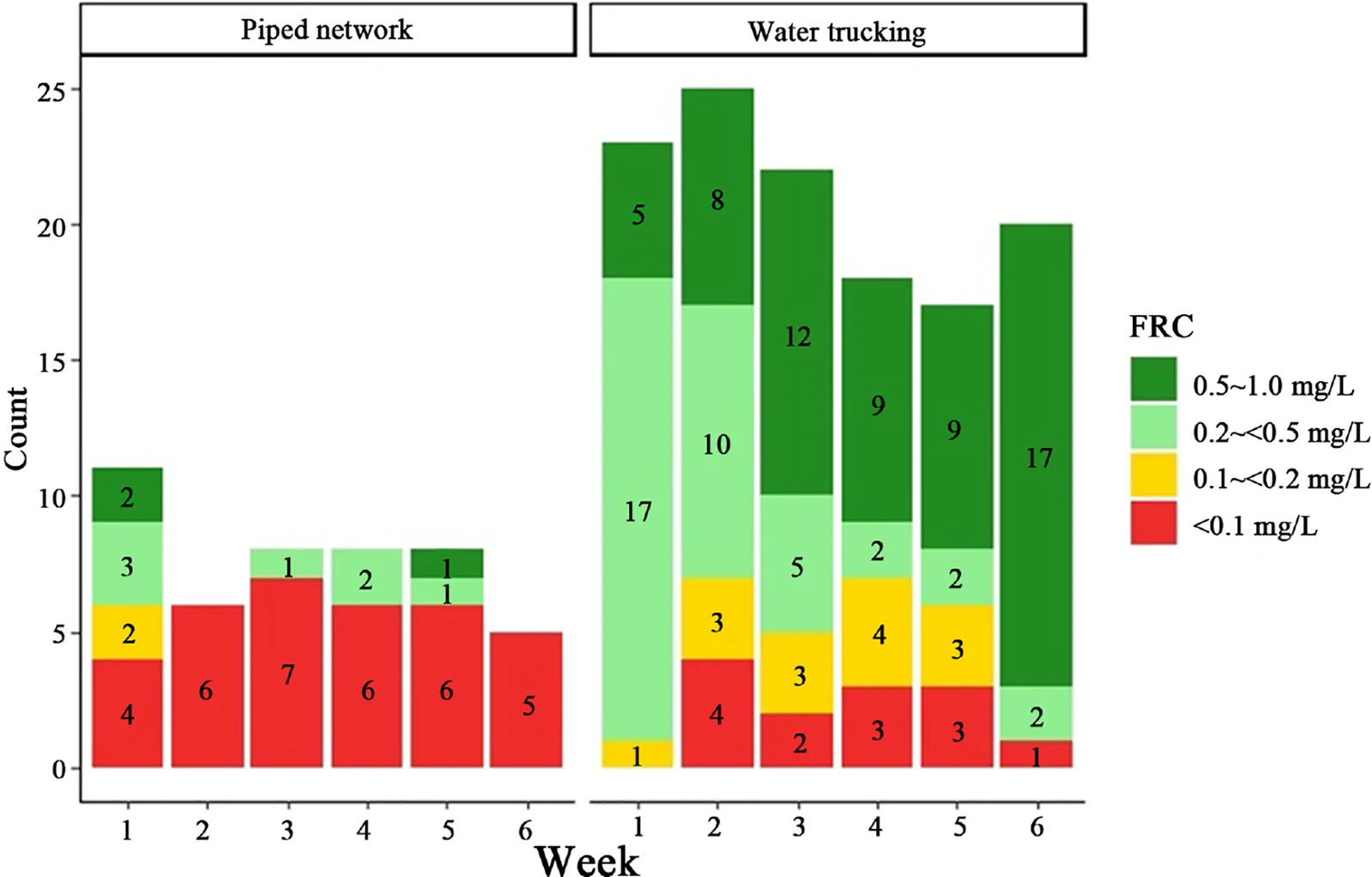
Free Residual Chlorine levels detected in accommodation centers by week and by water source in Beira, Mozambique.

The daily distribution of FRC levels detected in samples collected from the piped network is presented in Figure 2. Daily FRC levels in the piped network in Beira remained relatively consistent during the week, with lower FRC detected over weekends; 78% of weekend samples had detectable FRC versus 87% of weekday samples. At accommodation centers, 131 samples were tested from tanks filled by water trucks, 47 from new piped network points created at accommodation centers, and 6 from unspecified sources. The quality of water delivered by truck improved over time, however in those samples tested from the piped network that was extended to the accommodation centers, FRC levels remained low throughout the testing period; 26% of piped water samples had detectable FRC versus 90% of trucked water samples. The weekly distribution of FRC levels detected in accommodation centers is presented in Figure 3 by water source.

### 3.2. Pemba

**Figure 4.**
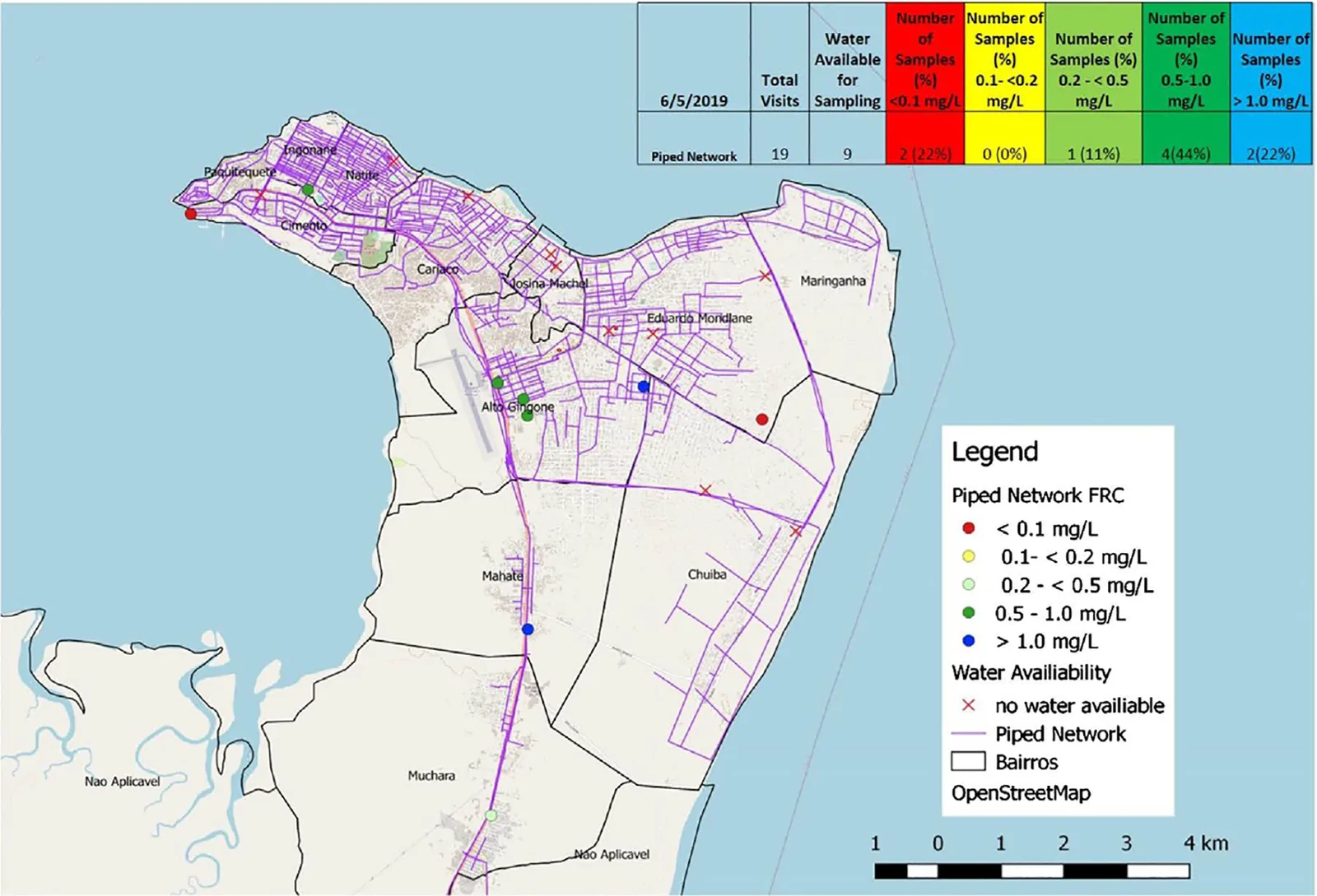
Example of a water quality monitoring daily report in Pemba. Summary of samples with free residual chlorine (FRC) testing data. Categories listed by FRC levels (mg/L). Map shows the spatial distribution of FRC in Pemba on June 5^th^, 2019.

In Pemba, water quality monitoring data collection began on May 30^th^. Figure 4 is an example daily water quality monitoring report shared with partners in Pemba. Overall, from May 30^th^ to June 20^th^, a total of 174 visits were made to the 30 sampling points selected on the piped network, and of these, 114 (66%) had water available at time of the visit; 64% of the 114 total samples collected had FRC ≥0.1 mg/L and 25% were within the recommended FRC range: 0.5 – 1.0 mg/L. The median FRC seen in the piped network was 0.2 mg/L and the range was 0.0 – 3.0 mg/L. Table 2 includes a weekly summary of all data collected between May 30^th^ to June 29^th^ in Pemba. Week 4 and 5 results are combined due to data collection stopping soon after the start of week 5.

## 4. Discussion

During a cholera outbreak, it is essential to provide safe water to at-risk populations as quickly as possible [1] [3]. In urban and peri-urban areas, existing piped networks may reach a significant proportion of the affected population; however, the quality of water can vary due to the length of the distribution network, intermittent flow, and breaks in the system [10] [11]. Water quality monitoring can help utilities manage their dosing to ensure that consistent chlorine levels are found at all parts of the network. Ensuring the quality of piped water may also be important in gaining the population’s trust in order to encourage use [12]. Setting up timely monitoring systems can be challenging during an outbreak or following a natural disaster. The monitoring systems set up in two cities of Mozambique demonstrate how rapid and simple water quality monitoring systems can be successfully mobilized in the early days of an emergency response. In Beira, 87% of the 1080 samples collected from the piped network had detectable FRC and 46% of the samples were in the recommended range. In Pemba, 64% of the 114 total samples collected had detectable FRC and 25% were within the recommended range. Daily water quality monitoring in Beira and regular water quality monitoring in Pemba were successfully integrated into the cyclone and cholera outbreak responses developed after cyclones Idai and Kenneth. This successful implementation illustrates that community-level chlorination improvements and water quality monitoring systems can be rapidly made and set-up to ensure adequately treated water is reaching affected communities.

Recent systematic reviews of water, sanitation, and hygiene (WASH) responses during cholera outbreaks and emergencies have highlighted that water quality interventions implemented during outbreaks and emergencies, particularly at the centralized level, such as those involving piped networks, have not been well-documented [2] [3] [13] [14]. Our work demonstrates that water quality monitoring programs can be implemented quickly and effectively during an outbreak. The use of mobile phones for data collection ensured data was quickly collected and analyzed for action. There was some added cost to the program with the purchase of phones and mobile data, however rapid data collection and collation provided actionable information quickly. The emergency water quality monitoring programs also allowed for the identification of several trends that helped the government and partners increase the effectiveness of the WASH response. First, the data identified areas in the piped network where FRC was consistently low and could be improved with the installation of booster chlorination stations. Certain points in the network, closely following distribution centers where boosting occurred, consistently had acceptable levels of FRC, whereas other areas, further away from these distribution centers had consistently low levels. The mapping and analysis of the water quality data allowed FIPAG, AURA, and DPS to identify sections where additional booster points could be added to the piped network. Second, inconsistent FRC data over time helped FIPAG identify areas with potential line breaks in the network that needed to be repaired. Third, the irregularity of FRC levels detected at certain points in the network highlighted issues with the consistency of daily treatment. In Beira, 78% of weekend samples had detectable FRC versus 87% of weekday samples. Staffing over the weekends was lower at piped network treatment stations, and the lower FRC levels detected over weekend days in the monitoring data allowed the utility to adjust weekend staffing and treatment. Fourth, regular data collection documented challenges with maintaining water quality levels in water tanks at accommodation centers. Accommodation centers received water from both the piped network and from water trucks that collected from boreholes and treated water during truck filling. At accommodation centers, the data identified gaps in the chlorination of piped water. Low FRC levels observed from the piped network may be because new connections were made to the network further away from distribution centers where chlorine boosting occurred. Fifth, in Pemba, water was often not available at the time of visit in locations that were scheduled by FIPAG to have water that day, which helped to identify locations that were not receiving water when scheduled. This also resulted in a reduced number of data points in Pemba.

Distribution of household water treatment products is common during outbreaks and has been shown to improve the microbiological quality of water when paired with appropriate training and follow-up by community health workers, to ensure that households understand the methods of treatment and when to use products [3] [13] [15]-[18]. However, it can take time for water treatment behavior change to occur at the household level and adherence to treatment has been documented to be challenging [19]-[21]. Household monitoring visits are often used to reinforce messaging, as households may choose not to use the tablets or use the incorrect dose [22] [23]. During the responses in Beira and Pemba, centralized chlorination of the pipe network was prioritized, and after the monitoring system was established, household water treatment products were provided to households that did not use the piped water supply. The advantages of this approach were that chlorinated water was provided to a larger population in a shorter amount of time and the limited household water treatment supplies could be prioritized for distribution in areas without access to the piped network. In addition, this approach allowed for a more efficient and simplified monitoring system where only sampling points on the piped network and at accommodation centers needed to be visited and more resource intensive household visits could be reserved for households that did not have access to the piped water supply. However, if this strategy is utilized, it is essential to continue to regularly monitor the piped supply to ensure that the water is treated and at an appropriate dose.

The following characteristics allowed the water quality monitoring programs in Beira and Pemba to be quickly established. First, partners were given access to already developed spatial maps of the piped networks to identify key points in the distribution network to include for sampling. Second, upon inception of the program, partners quickly agreed to use FRC as the primary parameter for monitoring with the understanding that the number of parameters could be expanded later. Lasty, mobile phone data collection methods allowed for the timely collection and dissemination of water quality data.

As part of this program, AURA, DPS, and FIPAG established weekly meetings where issues in the piped network could be addressed which allowed for collaboration between the three groups. As monitoring data stabilized, and FRC was detected at recommended levels at the majority of points in the monitoring system, a smaller number of points were prioritized once the water quality monitoring program was ready to transition from daily monitoring visits to visits twice a week. The water quality monitoring was a result of productive collaboration between government partners, FIPAG, CDC, UNICEF, the WASH Cluster and highlights the importance of coordination among agencies and building capacity at the provincial level. These activities could be reactivated in the event of a new emergency in Mozambique or replicated in similar emergencies in cholera endemic areas to prevent or control outbreaks.

## 5. Limitations

There were several limitations to this work. First, in both programs not all monitoring points were visited daily due to occasional staffing issues. Second, in Pemba, limited water availability meant that on certain days, water could not be sampled in parts of the piped network. Third, as accommodation centers opened and closed in Beira the number included in the program changed, making comparability at accommodation centers in some areas challenging. Fourth, chlorine readings from the chlorine test kits are based on a color comparator and individuals may interpret the color change differently. Supervision visits were included to ensure reliability of the data collected.

## 6. Conclusion

The results from this program indicate that water quality monitoring programs can be rapidly implemented in response to a humanitarian emergency and cholera outbreak to ensure safe water is reaching targeted populations. The water quality monitoring programs established after Cyclones Idai and Kenneth were a result of productive collaboration between government partners, CDC, UNICEF, and the WASH Cluster. Similar water quality monitoring activities could be replicated in other settings to improve chlorination through piped water systems in cholera endemic areas to prevent or control outbreaks. Use of mobile phones for data collection allows for the timely collation and dissemination of actionable water monitoring quality data.

## Figures and Tables

**Table 1.** Free residual chlorine (FRC) data collected from the water quality monitoring program in Beira from May 4^th^ to June 14^th^, 2019.

Location & Week	Total Visits	Water Available for Sampling	Number of Samples (%) <0.1 mg/L	Number of Samples (%) 0.1 – 0.2 mg/L	Number of Samples (%) 0.2 – 0.5 mg/L	Number of Samples (%) 0.5 – 1.0 mg/L	Number of Samples (%) >1.0 mg/L
**Piped Network Total**	1189	1080 (91%)	142 (13%)	126 (12%)	316 (29%)	495 (46%)	1 (0.01%)
**Week 1**	208	199 (96%)	18 (9%)	27 (14%)	70 (35%)	84 (42%)	0 (0%)
**Week 2**	221	173 (78%)	18 (10%)	24 (14%)	48 (28%)	83 (48%)	0 (0%)
**Week 3**	207	184 (89%)	17 (9%)	13 (7%)	58 (32%)	96 (52%)	0 (0%)
**Week 4**	201	177 (88%)	43 (24%)	16 (9%)	48 (27%)	69 (39%)	1 (1%)
**Week 5**	172	161 (94%)	18 (11%)	26 (16%)	48 (30%)	69 (43%)	0 (0%)
**Week 6**	180	173 (96%)	28 (16%)	20 (12%)	44 (25%)	81 (47%)	0 (0%)
**Accommodation Centers Total**	184	177 (96%)	47 (27%)	16 (9%)	45 (25%)	69 (39%)	0 (0%)
**Week 1**	37	37 (100%)	4 (11%)	3 (8%)	20 (54%)	10 (27%)	0 (0%)
**Week 2**	34	31 (91%)	10 (32%)	3 (10%)	10 (32%)	8 (26%)	0 (0%)
**Week 3**	30	30 (100%)	9 (30%)	3 (10%)	6 (20%)	12 (40%)	0 (0%)
**Week 4**	29	28 (97%)	9 (32%)	4 (14%)	4 (14%)	11 (39%)	0 (0%)
**Week 5**	27	25 (93%)	9 (36%)	3 (12%)	3 (12%)	10 (40%)	0 (0%)
**Week 6**	27	26 (96%)	6 (23%)	0 (0%)	2 (8%)	18 (69%)	0 (0%)

**Table 2.** Free residual chlorine data collected from the Pemba piped network from May 30^th^ to June 29^th^.

Location	Total Visits	Water Available for Sampling	Number of Samples (%) <0.1 mg/L	Number of Sample (%) 0.1 – 0.2 mg/L	Number of Samples (%) 0.2 – 0.5 mg/L	Number of Samples (%) 0.5 – 1.0 mg/L	Number of Samples (%) >1.0 mg/L
**Piped Network Total**	174	114 (66%)	41 (36%)	0 (0%)	31 (27%)	28 (25%)	14 (12%)
**Week 1**	95	59 (62%)	19 (32%)	0 (0%)	11 (19%)	16 (27%)	13 (22%)
**Week 2**	37	23 (62%)	7 (30%)	0 (0%)	9 (39%)	6 (26%)	1 (4%)
**Week 3**	21	17 (81%)	7 (41%)	0 (0%)	5 (29%)	5 (29%)	0 (0%)
**Week 4 – 5**	21	15 (71%)	8 (53%)	0 (0%)	6 (40%)	1 (7%)	0 (0%)
